# Current Perspectives on Paradental Cyst: A Literature Review

**DOI:** 10.3390/dj11120281

**Published:** 2023-12-05

**Authors:** Pei Li, Yifan Zhao, Yuehua You, Ling Lin, Dongsheng Yu, Wei Zhao

**Affiliations:** 1 Guangdong Provincial Key Laboratory of Stomatology, Guanghua School of Stomatology, Hospital of Stomatology, Sun Yat-sen University, Guangzhou 510055, China; lipei25@mail2.sysu.edu.cn (P.L.); linling23@mail2.sysu.edu.cn (L.L.); 2 School of Stomatology, Department of Medicine, Changsha Medical University, Changsha 410219, China; zhaoyifan721@163.com; 3 Department of Stomatology, Longhua People’s Hospital Affiliated to Southern Medical University, Shenzhen 518109, China; youyh23@mails.tsinghua.edu.cn

**Keywords:** cyst, jaw cyst, paradental cyst, buccal bifurcation cyst, cone-beam computed tomography, periodontitis

## Abstract

Paradental cyst (PC) is an uncommon type of odontogenic cyst of inflammatory origin, which develops near the cervical margin of the outside of the root of a vital tooth. The category of paradental cyst includes the buccal bifurcation cyst, which is found in the buccal area adjacent to the mandibular first or second molars in children. A conclusive diagnosis of a PC needs to correlate the surgical, radiographic, and histologic findings. When strict diagnosis is neglected, they can be easily misdiagnosed and mistreated. PCs associated with mandibular first and second molars and those associated with the mandibular third molar may have slightly different clinical manifestations but have almost completely different treatment principles due to the distinction in location. For the third molars, removal of both the tooth and the cyst is preferred. However, when the first or second molars are affected, it may be advisable to perform enucleation of the lesion while preserving the associated tooth. There are also more conservative methods to retain vital permanent teeth within the mandibular arch. Additionally, the cyst wall primarily consisted of granulation tissue firmly attached to the periodontal ligament space. The exact origin of these cysts was a subject of ongoing debate, but they were believed to primarily arise from either the reduced enamel epithelium or the inflammatory proliferation of junctional/sulcular epithelium, which originate from the superficial mucosa during tooth eruption. The aim of the present review was to update information on clinical manifestations, diagnosis and treatment strategies of cysts and discuss their pathogenic mechanisms. Raising familiarity with the distinctive features is beneficial for accurately diagnosing these lesions and effectively caring for the patients.

## 1. Introduction

According to the World Health Organization (WHO) classification [1], the term “paradental cyst” is a cyst that forms near the cervical margin on the lateral side of the root due to an inflammatory process within the periodontal pocket. A unique type of paradental cyst appears on the buccal and distal surfaces of emerged mandibular molars, typically the third molars, associated with a history of periodontitis. Paradental cysts are relatively rare in clinical practice, with an incidence of about 0.9% to 4.7% [2]. In fact, their true incidence may be much higher because their unclear clinical characteristics can easily lead to misdiagnosis. Moreover, it is easy to lose sight of buccal bifurcation cyst (BBC), a type of paradental cyst that typically develops beside mandibular first and second molars in juveniles, greatly affecting the development of teenagers’ teeth, jawbones and occlusion [1].

Patients with PCs often suffer from recurrent local pericoronitis with pain and swollen gums. Radiographically, cystic lesions can be seen on the distal side of the teeth or overlying the roots. When strict diagnosis is neglected, they would be easy to be treated as apical periodontitis or pericoronitis. Accurate diagnosis depends on the combination of clinical examination, imaging examination and pathological examination. To date, paradental cysts are still unrecognized by many clinicians. The etiology of PC is still vague and there is no consensus on the treatment plans. This premise poses a need for a detailed review. By referring previous studies, our article reviews the terminology, etiology, clinical presentation, diagnosis and differential diagnosis, treatment strategies and prognosis of paradental cysts in details to provide a comprehensive and up-to-date knowledge of paradental cyst. Therefore, we hope this review can serve as a tool to foster more adept and rational approaches to the diagnosis and management of paradental cysts, ultimately ensuring optimal patient care.

## 2. History of Terminology

As early as 1930, Hofarth et al. [3] had described multiple cases of a jaw cyst found distal to mandibular third molars with clinical indications of pericoronitis and dubbed it “marginal wisdom tooth cyst” according to a thorough literature review. Hofarth’s clinical, radiological, and histological description meets the current definition of a paradental cyst [2]. However, it was not until 1970 before scholars detailed the clinical and histological features of paradental cysts [4]. Then, the clinicopathological characteristics were described by Craig et al. [5] in 1976 when he proposed the term “paradental cyst”. In 1983, Stoneman and Worth [6] first illustrated the clinical manifestation of mandibular infected buccal cyst of the mandibular first and second molars, also named as “buccal bifurcation cyst” by some authors [7,8,9]. In 1992, the World Health Organization (WHO) incorporated BBC into their histological classification of odontogenic tumors under the designation of “paradental cyst”, identifying it as the “mandibular infected buccal cyst” [1]. It is worthy to clarify that BBC develops more commonly on the buccal side of the first and second mandibular molars of children [10,11], while classic PC on the third molars. Classic PC and BBC may share common etiology and microscopic features, so they are currently considered to be the same inflammatory entity that occurs in different sites [12]. However, due to the different tooth involved and the influence of adjacent anatomical structures, their clinical presentations, imaging features, and treatment methods are different [11]. Therefore, there is currently no consensus on whether the BBC is an independent disease of periodontal cysts. Throughout the history, many other terminologies like inflammatory paradental cyst [2,13,14,15], inflammatory lateral periodontal cyst [16] and inflammatory collateral cyst [4,17] were used. Slater [18] believed that eruption pocket cyst or pericoronitis-associated eruption cyst can better stress the clinical features of the cyst strongly associated with developing teeth. Among them, inflammatory paradental cyst and paradental cyst are the most widely propagated. In order to avoid confusion, paradental cyst is used throughout this article.

## 3. Epidemiological Features

**Figure 1:**
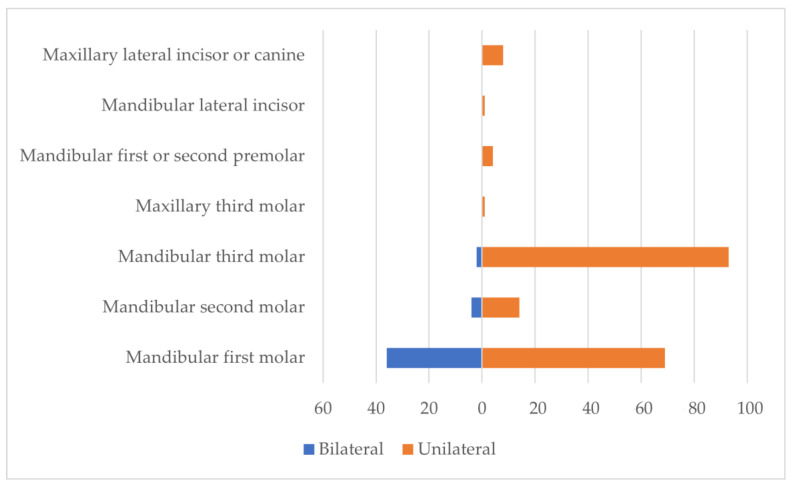
Distribution of unilateral and bilateral cases of paradental cysts.

Table 1 listed the epidemiological and clinical characteristics as well as treatment of PCs in the cases reports categorized by their tooth sites. Among the BBC cases, most BBCs (85.37%) have been linked to mandibular first molars, and 34.29% of which were bilateral cases. A small number of reports on BBCs (14.63%) were about mandibular second molar, with a small proportion of bilateral cases (22.22%). The incidence of BBC is much higher in the mandibular first molar than in the mandibular second molar. And bilateral location rate for mandibular first molar is higher than mandibular second molar (Figure 1). The remaining cysts were mostly associated with the mandibular third molars, and only 2 rare bilateral cases were reported (Figure 1). Individual cases were related to maxillary teeth and mandibular premolars [2,13,15,19], and no bilateral case was found (Figure 1).

When it comes to age distribution, the majority of BBCs involving the mandibular first molar occur between the ages of 6 and 10, while those involving the mandibular second molar occur between the ages of 9 and 15 (Table 1). Gender seems to have no apparent impact on the incidence rate (Table 1). Regarding the treatment methods, in cases involving third molars, the most common treatment methods are tooth extraction and cyst enucleation, while in cases involving other permanent teeth, the most employed treatment method is cyst enucleation with tooth preservation (Table 1).

## 4. Clinical and Radiological Features

### 4.1. Buccal Bifurcation Cyst

#### 4.1.1. Clinical Features

As for BBC, the mandibular first molars are the most frequent location, and in certain cases, the second mandibular molars might also be impacted. Jajam et al. [11] had reported an extremely uncommon case of maxillary first molar occurrence. The most common complaint is jaw swelling. In general, usually BBC have the following clinical features: (1) The cyst appears on the buccal side of the affected tooth; (2) The cyst grows near the eruption time of specific molar, that is to say, BBC of mandibular first molars are more common seen in 5–10 years old, and that of mandibular second molars are more common seen in 10–15 years old; (3) Recurrent buccal swelling; (4) Local discomfort and tenderness; (5) The cyst is associated with a vital tooth; (6) The crown of the involved molar may tilt buccally, with its apices approaching the lingual cortex because of cyst compression; (7) Deep periodontal pockets on the buccal side of affected tooth. Other symptoms and signs such as purulent discharge from the pockets [10,13], occlusal pain [26], and relative delay dental eruption [10,51,52] may present. Even in some cases, mild extraoral swelling as well as large palpable lymph nodes may occur, while others produced few symptoms and minimal signs which were found by chance [2]. Periodontal probing test is neglected in many articles probably because it may irritate pain and reduce the child’s cooperation. Bilateral symmetry exists in some circumstances and their size may not be the same. Sometimes symptoms only occur in one side. Accordingly, the other side should be examined for a second lesion even without a symptom [26,30,37,50].

Among all these examinations, most authors consider vital pulp (assessed by electrical viability testing or temperature testing) to be a very important diagnostic criterion [16,39]. Lim & Peck et al. [50] proposed that viability should not be a diagnostic criterion for BBC. In their report of paradental cysts of bilateral mandibular second molars, the left lower second molar was vital but the right one was nonvital. Meanwhile, their histological evaluation confirmed that their root pulp was fibrosed and the odontoblast layer was absent even with the coronal pulp showed normal. Yet later Slater et al. [18] questioned that in their histological section of the root pulp, the fibroblast nuclei and capillaries were intact and there was no evidence of necrosis, so there was a possibility of false negative pulp viability testing in the lower right second molar.

#### 4.1.2. Radiographic Features

**Figure 2:**
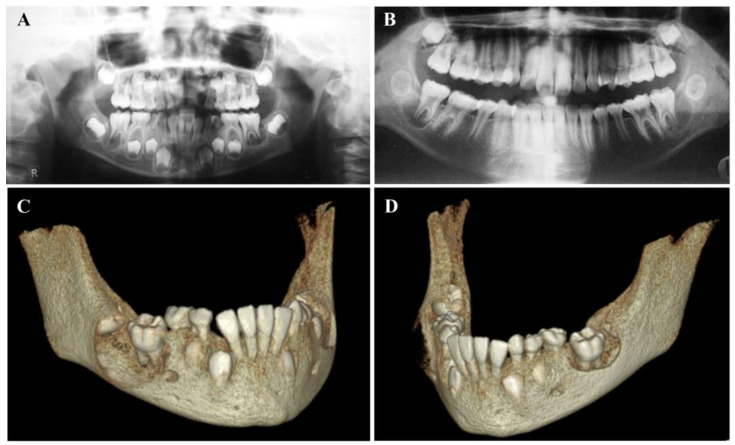
Panoramic radiographs and three-dimensional reconstructions in bone protocol of typical bilateral BBC lesions. (**A**). The panoramic radiograph displays distinct bilateral circular radiolucencies involving the erupting lower right first molar and lower left first molar. (Courtesy of Dr. Julio Corona-Rodriguez). (**B**). The panoramic radiograph shows bilateral radiolucencies associated with the lower left second molar and the lower right second molar. (Courtesy of Dr. Asher Ah-Tong Lim). (**C**,**D**) Three-dimensional reconstructions in bone protocol for the right (**C**) and left (**D**) mandibular regions highlight the lesions and their relationship with neighboring structures. (Courtesy of Dr. Cristhian Reynaldo Gomez Bautista).

Panoramic radiographs and apical radiographs are often used for routine examination, which can initially detect the presence of BBC. The former one is more convenient for initial screening of bilateral lesions (Figure 2A,B). Panoramic or apical radiograph reveals an ovoid radiolucency located at the bifurcation with clear outline, which can extend to the inferior border of mandible in the vertical direction, and reach over the mesial and distal root in the lateral direction [8,32,41]. Different from a periapical cyst, periodontal ligament space and lamina dura are continuous and intact, indicating that the lesion is not directly related to the root apices. Sometimes BBC may not be easily found due to the overlap of roots. A cross-sectional mandibular occlusal radiograph helps to exhibit buccal bony expansion and periosteal reaction (from monolayer to multilayered onion skin appearance), as well as displacement of compressed molars [20,32,51].

Cone Beam Computerized Tomography (CBCT) has had a major impact on maxillofacial imaging and has been widely used in clinic, which provides high-resolution as well as three-dimensional images. CBCT has been considered extremely useful in assisting diagnose and treatment for BBC. Although the radiation dose of CBCT is higher than that of plain films, it can display the contour of the cyst in the axial, coronal and sagittal planes. Three-dimensional reconstruction of the jaw emphasizes the findings and the relation of the lesion to the adjacent structures [10], which is conducive to accurate assessment and treatment (Figure 2C,D). Gallego et al. [26] used an interactive three-dimensional (3D) implant planning system not only to visualize the of cysts and their adjacent relationship (such as mandibular canal), but to evaluate the surrounding cortical bone thicknesses.

Additionally, Derindağ et al. [44] believed that ultrasonography (USG) was helpful in the aided diagnosis of BBC if a cyst produced a perforation in the buccal cortical bone and swelling in the soft tissue. They used an intraoral USG probe to exam the oral mucosa of the swollen area and found a lesion with submucosal localization and regular border. Most importantly, there is no radiation in the USG.

### 4.2. PCs of Mandibular Third Molars

#### 4.2.1. Clinical Features

In general, PCs of mandibular third molars usually appear after adulthood. Patients with have a history of pericoronitis with one or recurring spontaneous pain, swelling or chronic discomfort caused by occlusal trauma et al. [2,58,60,62]. Same with BBC, the vitality of the tooth was not in question and the tooth could be either partly or completely erupted. However, a communication from the periodontal pocket to the cyst is frequently found near the distal and bony expansion is not as common as BBC [2]. PCs usually present at the distal/distal buccal site of the third molars, less frequently at the mesial site and none have been reported at the lingual site [19,58,64]. Colgan et al. [58] raised a theory that there is a significant correlation between the location of PC and the angle of the impacted tooth because of the food flow, that is, the cyst lay mesially to mesioangularly impacted teeth, buccal to vertically impacted teeth and distal/distobuccal to the rest. Ozcan et al. [62] and Praksh et al. [61] reported extremely rare cases about PC occurring in supernumerary tooth fused with the mandibular third molar. The existence of a supernumerary tooth may have inhibited full eruption, resulting in cyst development [61].

#### 4.2.2. Radiographic Features

**Figure 3:**
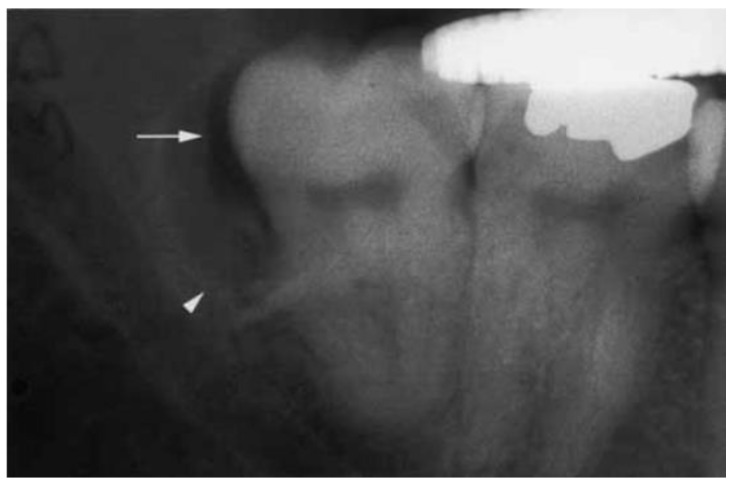
The periapical film illustrates the preservation of the follicle space (arrow) and the distobuccal location of the cyst surrounding the lower right third molar tooth (arrowhead). (Courtesy of Dr. Collette M. Colgan).

Radiographically, the borders of PC of mandibular third molar was more distinct than the border of BBC [65]. A well-defined, single-chambered, round radiolucency lesion often occurs on the distal or distal buccal side of the third molars. Sometimes, it displays semilunar radiolucency lesion superimposed onto the roots. They were usually about 10 ± 15 mm in diameter [2]. The periodontal ligament and the lamina dura are undoubted continuous. In addition, the preservation of space around the dental follicle (Colgan’s sign) (Figure 3) observed in some cases was considered to be a critical diagnostic indication to distinguish between PCs and dentigerous cysts, which suggested the dental follicle was not involved in the development of PC [58]. The WHO criteria pointed out that the cystic radiolucency of PC extended at least 4 mm beyond the follicle space of the tooth [16]. However, this image feature cannot be observed in the x-rays in many cases. Therefore, Colgan’s sign is critical but not widely applicable.

### 4.3. PCs of Other Teeth

Morimoto [13] collected 4 cases of PCs in mandibular premolars. Their clinical manifestations, imaging features and pathological features were accorded with the diagnosis of PC. In rare cases, PC was also reported to be associated with the maxillary anterior teeth region [14] and the palatal side of maxillary third molar [19].

## 5. Pathogenesis and Risk Factors

The etiology of PC is not fully clear and various theories have been proposed. It is currently thought that cyst formation may be associated with inflammation of the periodontal tissue around erupted tooth (pericoronitis) stimulating odontogenic epithelial cell proliferation [19]. The causes of inflammation may vary, and the epithelial origin is not fully understood. We currently attribute the triggers of infection to eruption factor, dental anatomy factor, and food impaction. There is not enough evidence to prove which is sufficient and necessary for the development of a PC. And according to the present study, it may be the result of a combination of factors.

### 5.1. Eruption Factor

**Figure 4:**
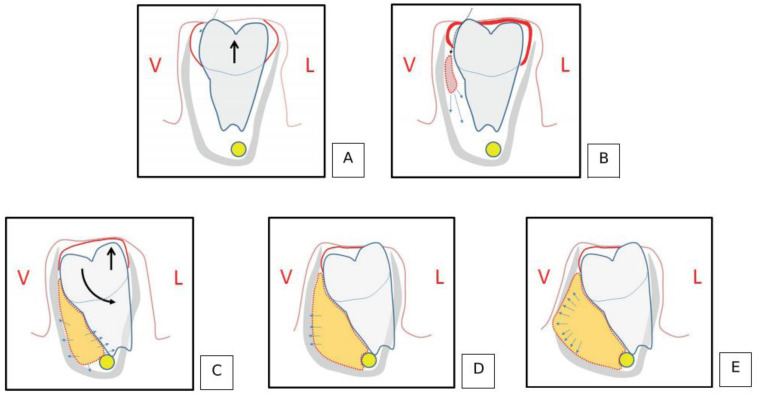
The development of a buccal bifurcation cyst at different stages in the theoretical hypothesis. (Arrows represent the direction of tooth movement). (**A**). The mesiobuccal cusp first breaks through the epithelium in the physiological eruption process. (**B**). The penetration creates a microscopic link between the pericoronal space and the oral environment, potentially causing localized inflammation in the epithelial attachment area. (**C**). The development of BBC leads to a tilted buccal crown and a raised lingual cusp. (**D**). BBC’s development results in cortical bone expansion. (**E**). BBC’s development causes cortical bone fenestration. (Courtesy of Dr. Aymeric Issler).

Many authors have proposed that in the process of tooth eruption, the epithelial attachment of connective tissue may appear inflammatory areas and stimulate epithelial proliferation when the mesiobuccal cusp first penetrates the oral epithelium (Figure 4). This theory may be supported by the fact that BBC often occurs on the buccal side, and the buccal tilting of the crown afterwards [2,29,33,38,51,66]. Only when a tooth is partially or fully erupted does the inflammation have a channel to get into the periodontal pocket, so clinically a BBC is often found during eruption. However, BBC does not occur during every eruption of the mandibular molar in every child. In this way, other contributing factors may be involved.

### 5.2. Dental Anatomy Factors

It was found that more than half of people may have cervical enamel projection (CEP) in their molars [67]. Connective tissues are unable to attach to CEPs, and therefore CEPs have been implicated as a risk factor for the development of localized periodontitis [68]. In the 28 cases of paradental cyst that Craig et al. [5] observed, 20 cases showed developing CEP or CEP extending from the enamel cementum to the bifurcation. The gingiva at the CEP is prone to the accumulation of bacterial plaque and development of inflammatory lesions during eruption [69], which can explain the buccal location of BBC. However, in most cases, the CEP were not seen in the involved tooth [64], so this factor is not decisive.

### 5.3. Food Impaction

Colgan et al. [58] reckoned that the inflammatory source of PCs in third molars may be due to food impaction, which lead to inflamed gingival and exudate accumulation in the swollen operculum, causing cystic dilatation by stimulating epithelial growth. The growth direction of the third molar affects the flow of food, thus curbing the location of cyst, as mentioned above. In addition, they extended this theoretical model to the occurrence of a BBC. The irregular-angle erupted mandibular first and second molars are prone to cyst formation. Food crowds on the buccal side of the teeth due to the buccal inclination when chewing, eliciting inflammation afterwards.

### 5.4. Epithelial Origin

The origin of the epithelial lining of PC has been debated for a long time but no direct evidence was found. The sulcular epithelium [50], cell rests of Malassez [69,70,71] or reduced enamel epithelium [5,58,69] are possible sources. The possible origin of sulcular epithelium is supported by the continuity of the cyst’s epithelial lining and the sulcular epithelium, but this could be due to irritation of sulcular epithelial cell proliferation after periodontal tissue breakdown [50]. Immunohistochemically, Maruyama et al. [19] examined ten surgical specimens of PCs and found that they were lined by epithelial cells displaying characteristics consistent with those of the junctional/sulcular epithelium. Craig et al. [5] suggested that cell rests of Malassez or reduced enamel epithelium may be the source. Nevertheless, Ackermann et al. [64] found that cell rests of Malassez in the lesions was not active. Additionally, the lining of PC should evenly enclose the root surface if the Malassez is the epithelial origin. At present, the reduced enamel epithelium is the most supported [58,64,71,72]. Serial sections of PCs of mandibular third molars indicated that cyst development may be accompanied by hyperplasia and cystic degeneration of reduced enamel epithelium. During tooth eruption, the reduced enamel epithelium takes on a more squamous appearance, which becomes more pronounced if eruption is blocked [66]. Meanwhile, its attachment to the enamel may be weaker so that pericoronal dental follicles connect to the oral cavity, resulting in a pericoronal pocket. The authors believed that the term pericoronal pocket, rather than periodontal pocket, best describes this condition [51].

In addition, symmetrical bilateral cysts account for a fair portion of reported cases. Shohat et al. [24] reported two cases which were identified as identical twins. Therefore, there may be local susceptibility factors or developmental causes [10,21,26,30,31,35,37,49,50], but the evidence is insufficient.

## 6. Pathology

### 6.1. Macroscopy

**Figure 5:**
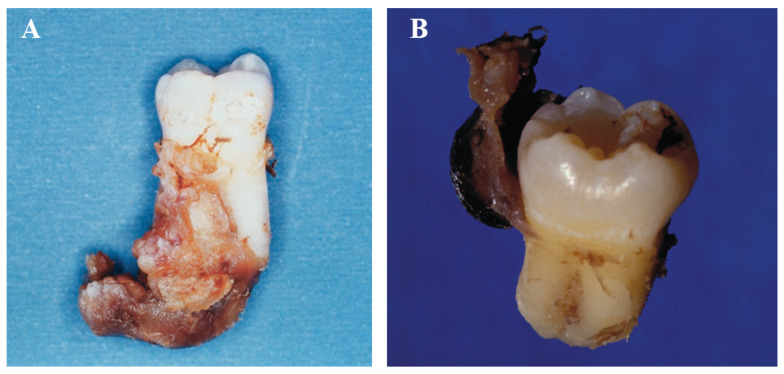
Macroscopic view of cysts attached to right mandibular first molar and third molar. (**A**). A macroscopic picture of the right mandibular first molar displays soft tissue positioned over the buccal bifurcation, extending distally and inferiorly towards radicular apices. (Courtesy of Dr. Tarcília Aparecida Silva). (**B**). A lingual picture of the lesion attached to the distal aspect of the extracted third mandibular molar reveals the relationship with the periodontal pocket. (Courtesy of Dr. Cláudia M. Kanno).

**Figure 6:**
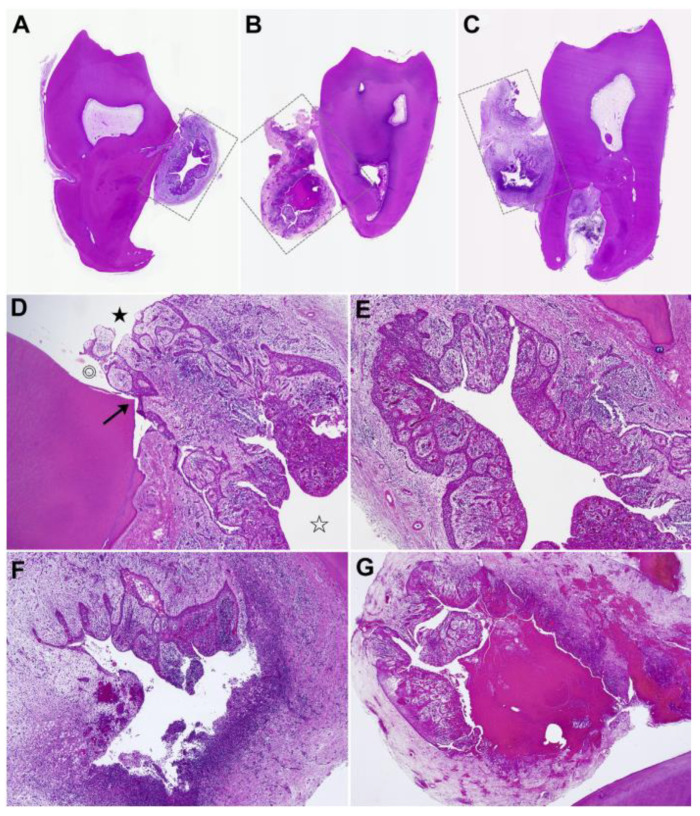
Histopathological features of paradental cysts at low-power view; H&E stain; (**A**–**C**), ×8, (**D**–**G**), ×25. (**A**–**C**). Three pathological samples, respectively, from three cases of paradental cysts. (rectangles representing the cysts). (**D**,**E**) Higher-power view of the cyst wall from (**A**). In the base of the periodontal pockets (double circle), there exists a transition zone (arrow) between the cyst lining the epithelium (open asterisk, cystic lumen) and the junctional/sulcular epithelium (closed asterisk). Cordlike anastomosing of lining epithelial cells is disrupted by granulation tissue along with a dense infiltration of chronic inflammation. (**F**). Higher-power view of the cyst wall from (**B**). Lining epithelia vanish in regions with intense inflammation. (**G**). Higher-power view of the cyst wall from (**C**). Prominent hemorrhagic tendencies with the cystic lumen occasionally contained bloody contents. (Courtesy of Dr. Satoshi Maruyama).

Most authors had similar descriptions of lesions in macroscopy. It was found that BBCs were attached to the CEJ without adhesion to any bone plate or the remaining roots, including the buccal region [9]. In Pompura’s case, the BBC cyst was tightly adhered from CEJ to bifurcation [8]. Silva et al. [51] described that the cyst extended from the buccal bifurcation to the distal and inferior apical region (Figure 5A). For third molars, cysts may similarly attach the CEJ to the bifurcation area or a certain part of the root [59,60,71] (Figure 5B and Figure 6A–C).

### 6.2. Microscopy

#### 6.2.1. Histopathological Findings

**Figure 7:**
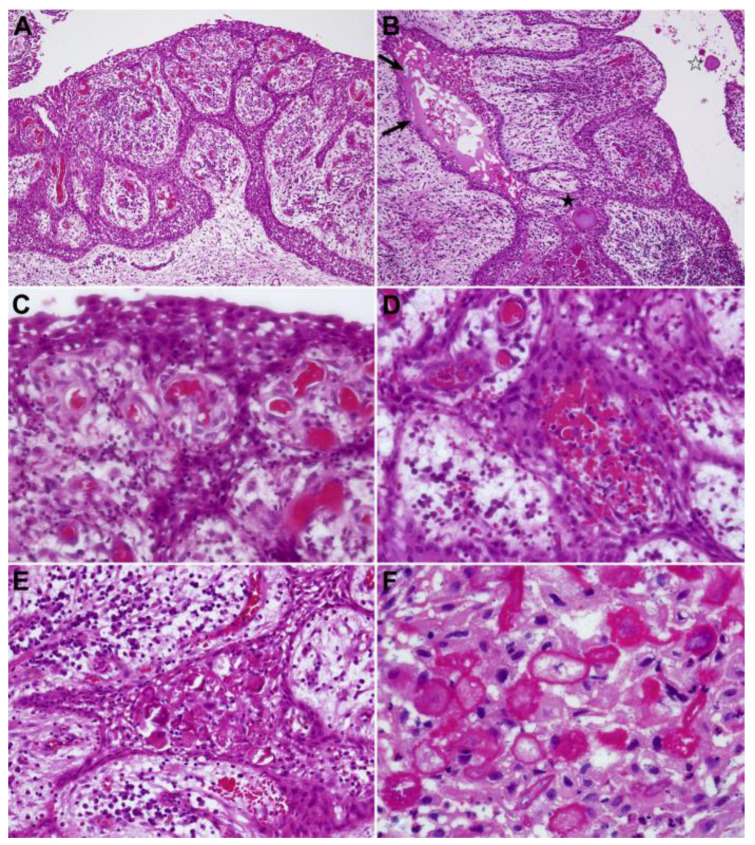
Histopathological features of paradental cysts at high-power view. (H&E) stain. (**A**,**B**) ×40, (**C**–**E**), ×160, and (**F**) ×300. (**A**). Significant congestion and vascular dilation of the blood capillaries can be seen in the immature granulation tissue separated by the anastomosed cords of lining epithelia. The intercellular space in the epithelial compartment exhibited distinctive widening. (**B**). Occasionally, microcystic alterations caused by the retention of tissue fluid, bloody contents, or certain foreign body-derived materials (arrows) can be seen, with macrophage reactions in the epithelial section (closed asterisk) or within the cystic lumen (open asterisk). (**C**). Red cells congested within the capillary space underwent coagulation or hemolysis. (**D**). Red cells extravasated within epithelial compartments or their intercellular spaces underwent coagulation. (**E**). Hyaline bodies in lining epithelia: eosinophilic, round-shaped materials occasionally developed within the epithelial compartment. (**F**). Hyaline bodies consisted of granular materials or enclosed with condensed borders. Some with nuclear traces or calcified materials in the center. (Courtesy of Dr. Satoshi Maruyama).

PCs are classified as odontogenic cysts of inflammatory origin, with a nonspecific inflammatory presentation resembling to those of radicular cyst [16], so similar findings can be seen in different tooth sites. We can use hematoxylin and eosin (H&E) staining and microscope observation to identify nonspecific inflammatory cysts. Figure 6 and Figure 7 shows the histopathological features of PCs of the mandibular third molars, which can also be used as a reference for the PCs arising from other teeth, such as BBCs. The fundamental component of the cyst wall is attached to granulation tissue aligning with the periodontal ligament area, with a keratinized stratified squamous epithelium-shaped spongy layer, and varies in thickness [19,73]. Large numbers of chronic or mixed inflammatory cells infiltrate the connective tissue of the cyst wall, where vascular expansion, hemosiderin pigment, cholesterol crystal fissures and foreign body giant cell reactions sometimes exist [13,19]. Using immunohistochemistry, Maruyama et al. [19] first found that the lining epithelia of PCs from the mandibular third molars exhibited positivity for K13, K14, and K19, along with perlecan and UEA-I binding, which differed markedly from the radicular cyst and dentigerous cyst. We need more research to confirm these results, and perhaps immunohistochemical staining can become the gold standard for distinguishing between PCs and other clinically similar cysts.

#### 6.2.2. Microorganism

PCs present with deep periodontal pockets, which contain mixed oral flora including α-Streptococci, β-Hemolytic streptococci, Staphylococcus epidermidi, Streptococcus pneumoniae, etc. [8]. Pelka et al. took [29] samples from the inflamed pocket for a DNA hybridization test in a BBC case to identify possible pathogenic microorganisms. Microorganisms of both Actinobacillus actinomycetem-comitans and the red complex were not detected. However, there were elevated levels of Fusobacterium nucleatum, a bacterium typically presented in a periodontal abscess. PC may not be extremely harmful or cause immediate damage to tooth eruption based on the findings. Therefore, the authors recommended against surgical intervention, suggesting that managing inflammation by opening and emptying the pocket could be enough to make the cyst disappear. There have been few microbiologic studies of PC. More evidence is needed to identify its microbial community characteristics (e.g., if there is an elevated concentration of some particular types of bacteria to help differentiate it from other inflammatory cysts), and therefore, a microorganism can be considered as an assistant diagnostic basis for PCs.

## 7. Diagnosis and Differential Diagnosis

### 7.1. Important References for Diagnosis

Overall, clinical and radiographic evaluations, as well as biopsy results, are all important to help make a diagnosis or exclusion [33]. A conclusive diagnosis was once made by WHO criteria combining the clinical, radiological, and histological results for each lesion [1]. We believe that PCs can be diagnosed according to the following points: (1) the tooth involved must be partially or fully erupted and in direct contact with the cyst; (2) the associated tooth must be vital; (3) a deep periodontal pocket can be found on the side where the cyst was attached; (4) a scan shows that the cyst exists near the CEJ area with a clear edge; (5) pathological examination reveals a non-specific inflammatory odontogenic cyst; (6) there is no other identifiable lesion such as giant cell granuloma, eosinophilic granuloma, dentigerous cyst, ameloblastoma or odontogenic keratocyst, that is to say, the histological features were not those of any recognizable histological entity, such as an odontogenic keratocyst or lateral periodontal cyst. In addition to the common features mentioned above, as a typical clinical feature for BBCs, age-specific and site-specific characteristic can to some extent help increase our vigilance against odontogenic cysts in these age groups.

Among them, vital pulp of the involved tooth has been considered a crucial diagnostic criterion for a long time. A pulp vitality test assessed by electrometrical testing, however, is sometimes difficult to carry out on an impacted third molar, or to obtain an accurate result on a young permanent tooth with a large apical foramen. Meanwhile, the temperature test is based on subjective consciousness, which cannot be considered the most scientific reference when it comes to children. Given that there is currently no perfect non-invasive technique for assessing pulp vitality for these situations, doctors need to combine other checks such as an intraoral examination (e.g., if there is the presence of predisposing factors for pulp inflammation), and an x-ray to make a comprehensive judgment.

### 7.2. Differential Diagnosis

The differential diagnosis of this lesion includes lateral radicular cyst, dentigerous cyst, eruption cyst, gingival cyst, periodontal abscess, pericoronitis, hyperplastic dental follicle, odontogenic fibroma, odontogenic keratocyst, and ameloblastoma. Among them, dentigerous cyst, lateral radicular cyst and PCs have many similar clinical and imaging manifestations, as well as pathological features, so they are described separately.

Dentigerous cysts can be developmental or inflammatory. The former one mostly results from the fluid exudation between the reduced enamel epithelium and the crown surface after the formation of the crown or root. The latter one occurs from the spread of previous periapical inflammation of deciduous tooth or from other sources to the dental follicle. The inflammatory dentigerous cyst would be suspected when mandibular premolar is involved, and the replaced deciduous molar is non-vital [13]. In addition, a dentigerous cyst presents as a radiolucent area around the crown of a non-erupted tooth, while PC arises on the lateral crown of an erupted tooth. Damante et al. [74] tried to established a connection between the radiographically measured pericoronal space width and the microscopic characteristics of the follicle, in order to enhance the diagnostic accuracy for small dentigerous cysts and paradental cysts. Ultimately, the definitive differentiation between them relies on clinical and surgical findings, such as the identification of bone cavities and the presence of cystic content. Bautista et al. [10] took CBCT as an important aid because buccal expansion and the lingual tilting of the apexes could be observed through different sections, which are typical features of PC instead of dentigerous cyst.

A radicular cyst can develop because chronic inflammation and the long-term stimulation of pulp cause proliferation of the rests of Malassez to form the cystic lining, accompanied by degeneration and liquefaction of the hyperplastic epithelial mass, along with continuous exudation of surrounding tissue fluid [75,76]. A viability test is an important diagnostic criterion for them. Radicular cyst would be the first consideration when tooth discoloration or painful percussion appears but without deep periodontal pocket. In the meanwhile, imaging analysis and viability test are inevitable [28]. Silva et al. [51] reported a rare case, where PC of the mandibular second molar approached the roots of the adjacent first molar, leading to its root resorption and loss of vitality. It could be easily misdiagnosed as a radicular cyst of the first molar. The author found that the cyst originated from the second molar during treatment, which was hard to find out initially based on screens and exams.

## 8. Recommended Treatment and Prognosis

To date, the choice of treatment depends on the specific tooth affected. For third molars, enucleation of PC with tooth extraction is the recommended treatment, while for the first and second molars, enucleation, marsupialization, or irrigation plus drug delivery in the periodontal pocket is more appropriate. The reported clinical characteristics of PCs and their corresponding treatment may provide reference for clinical practice (Table 1).

The current treatment regimen is becoming more conservative, and non-surgical methods seem to have positive effect in the short run, which is a positive development. However, surgical treatment is advised if continuous expansion or infection occurs, in order to avoid severe conditions such as root resorption or mandibular nerve canal compression. Therefore, there is a need for more clinical studies investigating the long-term outcome of the conservative treatment to inform clinicians about implications for medical intervention. Before conservative treatment, doctors should explain various situations to patients and their parents, including affected teeth extraction in the final.

### 8.1. Enucleation Plus Tooth Extraction

In general, the third molar is of little value to retain, so removal of the third molar tooth with curettage of the PC is the most popular choice [2,14,58,61,65,70]. New bone formation is spontaneously in the extraction wounds and no recurrence cases have been reported. In special occasions that involve root resorption or extreme malposition of mandible first or second molar, tooth extraction would be a consideration or sometimes inevitable [51,52]. Annibali et al. [23] considered that the involvement or non-involvement of the dental papilla and papillary lamina dura is the basis for determining the treatment. If these structures are implicated, extraction is advisable in the case of an impacted tooth.

In Heggendorn’s case [55], a large bone defect was formed, resulting from the enucleation of PC, and Leukocyte-platelet-rich fibrin (PRF) was used for maintenance of the bone volume. They reckoned that the physical, chemical, and biological properties of the bone substitutes might enhance rapid bone formation when bone grafts were combined with PRF. Radiological evaluation showed new bone formation after 40 days, indicating that it had osteogenic and osteoinductive effects. This approach can be considered in cases of extensive bone defects.

### 8.2. Enucleation without Tooth Extraction

Enucleation of the cyst without tooth extraction is broadly recommended for BBCs. Undoubtedly, the loss of first or second molar in a growing child will significantly affect the overall development of occlusion and therefore we should try to keep it if possible. If the lesion can be completely removed, it will rarely recur and bone healing is achieved; thus, the tooth can erupt further. Until now, cases that have applied enucleation of the BBC alone with preservation of the associated tooth were followed up for 0.5 to 3 years [25,34,35,36,37,38,39,40,41,42], and there were no returns and no need for reoperation or tooth extraction. Additionally, Gallego et al. [26] reported that an interactive 3D implant planning system can better assist enucleation, which showed clear and readily visualizable view of the cyst and its relation with the molar and the neighboring neurovascular features.

To deal with large bone loss after enucleation, Levarek et al. [41] used bone grafting as a treatment adjunction as well. Their patients received a bone graft immediately after enucleation of the cyst or secondarily, which turned out to enhance bone regeneration, provide stability and adequate root and furcation coverage of the involved tooth, and reestablish the alveolar crest to the level of the cementoenamel junction.

It is currently the predominant treatment approach for BBC (Table 1), enjoying broad application and ensuring therapeutic efficacy. Compared with other treatments, the one-time curettage of cysts without tooth extraction under local anesthetic may be performed in the absence of worry, with minimal pain and swelling postop. However, thorough removal of the cyst may jeopardize the periodontal support of the afflicted tooth, and surgical treatment has complications such as the loss of bone tissue, damage to blood vessels and nerves and the possibility of damaging adjacent anatomical structures. Those are the major downsides of the surgical method.

### 8.3. Marsupialization

The surgical marsupialization of BBC was reckoned to lead to a good outcome without compromising the development of the related tooth, and complete periodontal healing was achieved [33]. In their procedure, marsupialization left the cyst cavity in communication with the oral environment, remaining exposed to allow spontaneous osseous regeneration. Yet the long-term care of keeping the surgical area clean requires a high cooperation of patients and their parents. In case of poor compliance, enucleation should be considered.

### 8.4. Other More Conservative Treatments

It seemed that some BBC cases appeared to be “self-limiting” [22]. Some occasional microtrauma, including periodontal exploration, may cause the cyst to decompress and then heal spontaneously. However, there is no clear indication when observation or surgical treatment is required, and it is hard for clinicians to give no disposal when large cysts cause severe bone resorption and greatly affect the developing teeth.

Pelka et al. [29] combined the minimally invasive surgical drainage of the pocket and the sustained release of 10% doxycycline hydrochloride gel to rapidly improve painful and swelling symptoms. Subsequently, only cleaning was required, without any repeated topical antibiotic treatment. After 2 years, the BBC completely disappeared. In addition, David et al. [7] raised that more conservative procedure was enough for treating BBC. They used saline and hydrogen peroxide to irrigate the pockets and the patient was instructed to repeat at home daily with saline rinses. The lesion was resolved with irrigation and the periodontal status returned to normal after several months. These treatments may be appropriate for children with dental fear or those with poor cooperation in hospital. Non-invasive treatment is also indicated for children with underlying conditions that require the careful consideration of surgery.

## 9. Conclusions

Generally, most authors have agreed that BBC is an inflammatory paradental cyst. They attributed their variation of clinical presentations between children and adults to diverse host responses under infection. Through years of research, we now have a deeper understanding of the formation of a PC, which is triggered by the erupting tooth with pericoronitis in particular. The cyst-attached tooth must have partially or fully erupted, otherwise the incentives outlined above have no access to induce inflammation. This type of inflammation that may not necessarily be self-eliminating may threaten the growth and development of young permanent teeth and the establishment of occlusion in teenagers. So, it is crucial for clinicians to understand the most common features, correct diagnosis, and treatment methods of this entity. As more conservative treatments become prevalent, it seems that we have multiple choice to treat PCs with the good results. On the condition that no recurrence is reported up to now, we can easily draw a conclusion that PCs have a promising treatment outcome.

The number of publications of PCs is still small, but it is gradually expanding with clinician’s increasing awareness. However, there are many problems we have not solved. For example, the exact etiology and underlying triggering factors of PCs are still not clear and therefore preventive strategies are unknown. Lastly, more research of PCs should be reported in the future as to figure out the etiological mechanism and prognosis assessments of them, hopefully laying the foundation for the development of clinical pathway.

## Figures and Tables

**Table 1:** General characteristic of paradental cysts in the literature since 1990.

Tooth Involved	Authors (Year)	*n* of Cases	Gender	Age (Years)	Treatment	Follow-Up (Months)
Mandibular first molar	Packota et al. (1990) [20]	5 (1 bilateral)	ND	6–8	En	6–9
	Bohay et al. (1992) [21]	1 bilateral	M	7	En	8
	Pompura et al. (1997) [8]	32 (12 bilateral)	18 F 14 M	5.5–11	En	30–36
	David et al. (1998) [7]	3 bilateral	M	7–9	Surgical treatment (right, ND) and SR (left); SR; PI	9–15
	Gomez et al. (2001) [22]	1	M	10	SR	8
	Annibali et al. (2002) [23]	1 bilateral	F	7	Ma (left) and En (right)	72
	Shohat et al. (2003) [24]	3 (1 bilateral)	2 F 1 M	8	Ex; En	24–36
	Philipsen et al. (2004) [2]	1	M	47	En	ND
	Lacaita et al. (2006) [25]	10 (3 bilateral)	7 F 3 M	6–9	En	24
	Gallego et al. (2007) [26]	1 bilateral	M	8	SR (right) and En (left)	12
	Iatrou et al. (2009) [27]	4	M	7–9	Ex, En	ND
	Thikkurissy et al. (2010) [9]	1	M	7	En	14
	Borgonovo et al. (2010) [28]	2	M	7, 8	En	12
	Pelka et al. (2010) [29]	1	M	7	PI and topical antibiotics	24
	Corona-Rodriguez et al. (2011) [30]	1 bilateral	M	7	En (right) and SR (left)	6
	Zadik et al. (2011) [31]	1 bilateral	ND	7.5	SR	ND
	Chrcanovic et al. (2011) [32]	1	F	6	En	16
	Lizio et al. (2011) [33]	5 (1 bilateral)	3 F 2 M	7–10	Ma	12–60
	Santos et al. (2011) [34]	1	ND	8	En	6
	Borgonovo et al. (2012) [35]	1 bilateral	M	8	En	12
	Boffano et al. (2012) [36]	1 bilateral	ND	9	En	6
	Ramos et al. (2012) [37]	1 bilateral	M	9	En	12
	Issler et al. (2013) [38]	2 (1 bilateral)	1 F 1 M	9	En	2–18
	Borgonovo et al. (2014) [39]	1	M	6	En	ND
	Friedrich et al. (2014) [40]	1	M	6	En	15
	Levarek et al. (2014) [41]	3	2 F 1 M	6–7	En	5–9
	Kim et al. (2018) [42]	2	M	8, 9	En	6, 24
	De Grauwe et al. (2018) [43]	3	1 F 2 M	6–8	En, Ex	24
	Derindağ et al. (2019) [44]	1	M	10	En	ND
	Bautista et al. (2019) [10]	1 bilateral	F	7	En	ND
	Lima et al. (2019) [45]	1	M	7	En	7
	Aloyouny et al. (2020) [46]	1 bilateral	M	7	Ex	12
	Dave et al. (2020) [47]	3 (2 bilateral)	2 F 1 M	6–11	En, Ex	6
	Ruddocks et al. (2022) [48]	8 (2 bilateral)	3 F 5 M	8–9	En, ND	5, ND
Mandibular second molar	Martinez-Conde et al. (1995) [49]	1 bilateral	M	11	Ex	ND
	Lim et al. (2002) [50]	1 bilateral	F	13	Ex	ND
	Lacaita et al. (2006) [25]	2	1 F 1 M	9	En	24
	Shohat et al. (2003) [24]	2 (1 bilateral)	1 F 1 M	11, 13	Ex, En	24–36
	Silva et al. (2003) [51]	1	M	14	En	ND
	Philipsen et al. (2004) [2]	4	1 F 3 M	12–40	En	ND
	da Graça et al.(2004) [52]	1	F	15	Ex	ND
	Borgonovo et al. (2013) [53]	1	F	14	En	6
	Maruyama et al. (2015) [19]	1	M	17	En	ND
	Oenning et al. (2018) [54]	2	1 F 1 M	13, 17	En	ND
	Heggendorn et al. (2021) [55]	1	F	14	Ex, bone regeneration	8
	Ruddocks et al. (2022) [48]	1 bilateral	1 F 1 M	11, 12	ND	ND
Mandibular third molar	Lindh et al. (1990) [56]	1	M	21	Ex	ND
	Costa et al. (2014) [57]	55	ND	ND	Ex	ND
	Colgan et al. (2002) [58]	15	8 F 7 M	18–43	Ex	ND
	Philipsen et al. (2004) [2]	12 (2 bilateral)	5 F 7 M	18–46	Ex	ND
	Kanno et al. (2006) [59]	2	2 M	21, 23	Ex	ND
	Mufeed et al. (2009) [60]	1	F	21	Ex	ND
	Prakash et al. (2012) [61]	1	F	28	Ex	ND
	Maruyama et al. (2015) [19]	6	4 F 2 M	22–47	Ex, En	ND
	Ozcan et al. (2016) [62]	1	M	27	Ex	ND
	Vasanthi V (2023) [63]	1	M	23	Ex	1
Maxillary third molar	Maruyama et al. (2015) [19]	1	M	56	Ex	ND
Mandibular first or second premolar	Morimoto et al. (2004) [13]	4	3 F 1 M	9–10	En	ND
	Maruyama et al. (2015) [19]	1	M	45	En	ND
Mandibular lateral incisor	Maruyama et al. (2015) [19]	1	M	65	En	ND
Maxillary lateral incisor or canine	Vedtofte et al. (1989) [15]	8	1 F 7 M	10–49	En	ND

ND: Not in detail. En: Enucleation with tooth preservation. M: Male. F: Female. SR: Self resolution. PI: Periodontal irrigation. Ma: Marsupialization. Ex: Tooth extraction with cyst enucleation.
